# The Birmingham Hospital Saturday Fund

**Published:** 1894-06-09

**Authors:** William T. Smedley


### EDITOR'S LETTER-BOX.

[Our correspondents are reminded that proxility is a great bar to publication, and that brevity of style and conciseness of statement greatly facilitate early insertion.]

### THE BIRMINGHAM HOSPITAL SATURDAY FUND.

MR. W. T. SMEDLEY, the hon. secretary, has sent us a statement much too long to publish in its entirety, and as his condensation did not reach us in time, we give the material portions of it in Mr. Smedley's own words:—

I regret having to trouble you with any further remarks upon this subject; but the article which appears in your issue of May 26th is so thoroughly unfair, that I am compelled to ask you to give me space to reply thereto.

After a general statement as to the services rendered by a number of persons in addition to those mentioned by Mr. Burdett, Mr. Smedley proceeds:—

I now refer to your criticisms on my assertion:—

Then (in 1878) were the original foundations of the movement removed and others substituted. Building operations were commenced on altogether different lines. Mr. Gamgee recognised this and protested. He spoke and wrote against the policy adopted by the movement. On July 27th, 1882, he delivered a public address, in which he strongly criticised the altered conditions under which the collection was conducted. Mr. Sampson Gamgee was undoubtedly the founder of the movement in Birmingham, but whatever success it has attained to-day has not been built on the foundations he laid, but on foundations laid in direct opposition to his advice.

You meet me with a direct negative on these points, and say that as I have appealed to Cæsar, there is no alternative left but to examine Cæsar's judgment. I absolutely and unhesitatingly re-assert every word that I have quoted. It appears to me incredible that, having Mr. Gamgee's address in your hand, with a vestige of regard for the fair treatment of an opponent you could deny the truth of my assertion. Nothing can be stronger than the testimony of the address to the truth of my position.

The quotation which you have given and represented as Mr. Gamgee's views on the weekly system contains only the admission he was compelled to make in its favour *after its success was assured, that is in,* 1882, whilst by inference condemning the Committee for recommending it, and whilst avowing that the weekly system formed no part of his original Hospital Saturday scheme.

Mr. Gamgee says—

The complete success of Hospital Saturday depends in no small measure upon the method of collecting the money, and this is a matter attended with no little difficulty. Acting on the experience gained with the Working Men's Fund for the extension of the Queen's Hospital, our Committee abstained most scrupulously from recommending any set method of collection. The recent incident which resulted in a workman recovering in a Court of Justice money said to have been stopped at a penny a week for Hospital Saturday is only recalled to point to a possible danger attending the system. The projectors of Hospital Saturday did not aspire to make it a success on the mere mechanical principle of "many a mickle makes a muckle." The penny-a-week system in factories and workshops is vastly older than Hospital Saturday, in instituting which it was hoped to sanctify a labour day, by the sacrifices and loving thoughts of those yet blessed with the strength to work on behalf of fellow-toilers laid low by injury or disease, &c.

That is Mr. Gamgee's definition of to "make the day itself a day of labour sacrifice." You say this "is of course to contribute towards the cost of the free work of the hospitals." Why Mr. Gamgee's object—avowed object—in delivering his address, was to tell us that in so interpreting it we were tearing up the foundations he had laid! "The penny-a-week system in factories and workshops is vastly older than Hospital Saturday, in instituting which it was hoped to sanctify a labour day." A magnificent conception! With Mr. Gamgee preaching it, a potent force, in 1873, a year of unexampled good trade, when men were in full work, and masters were glad for them to work overtime upon any pretence! Without Mr. Gamgee's inspiring power, a hopeless failure in 1878, with bad trade, and workshops working on short time only three or four days a week! Mr. Gamgee told me again and again—he told the few members of the 1873 Committee, who had not deserted us, again and again—that by recommending the weekly system of contributing, we were undoing everything he had sought to accomplish. The collection, which in 1873 produced £4,705 11s. 3d., and had fallen in 1878 to £3,134 5s., had, by the aid of weekly contributions, risen in 1882 to £4,888 18s. 9d.

Within two or three days after the division of the money, in June, 1892, Mr. Gamgee met me, congratulated me upon the success, and went on to say that the Hospital Saturday Fund had become so changed that he felt it his duty to put on record what was the foundation of the movement as he laid it, or that it had ever existed might be forgotten, and he should take an early opportunity of doing this. On the 27th of the following month he carried out his intention. My quotations have been made from a copy of that record bearing my name in his handwriting, marked "with the author's compliments."

The ideal of his Hospital Saturday was far and away before ours, but ours is the practical, though less poetic, method of effecting the same ends.

In quoting from the address certain objects for good work to stimulate exertion in their support, you say:—

The gift to some existing institution by the working classes of an accident ward, an operating theatre, a convalescent hall, or invalid baths, *an extension of the sanatorium at Blackwell,* and for the Hospital Saturday Fund to make arrangements on the co-operative principle, to send some of Birmingham's sick toilers to the convalescent homes at Bath, &c.

The italics are not Mr. Gamgee's, and I presume they are intended to suggest that the words may be taken to refer to some such a step as the establishment of our convalescent homes at Llandudno. But there is this essential difference, the key to which lies in the words you use, "to some existing institution." Mr. Gamgee never contemplated the working men establishing and administering such institutions themselves, but only as providing the funds for others to administer. Nothing that he has written points to his considering such a course possible. That idea is not his.

The remarkable fact that for 16 years there has been a steady annual increase in the amount collected and distributed as a free gift by the working men of Birmingham—from £3,134 in 1878 to £11,916 19s. 6d.—points to something else besides foundations as the cause of success. It is that we have traversed the only sure road to success by constant vigilance and unremitting labour in organisation.

Yesterday was handed to me a Hospital Saturday workmen's balance-sheet, typical of upwards of a thousand which have been prepared during the past week here. It is that of the road men (menders and scavengers) employed by the Corporation. They collected £94 15s. 8d., to which was added 6s. 8d. for bank interest, and £19 16s. 3½d. brought from last year; total, £104 18s. 7½d. They bought 202 Dispensary tickets, £28 7s.; 39 General Hospital notes, £6 16s. 6d.; 15 Eye Hospital, £1 11s. 6d.; 6 Ear and Throat Hospital, £1 1s; and paid £3 12s. 6d. for registration fees at the Queen's, Children's, and Women's Hospitals, absorbing £81 8s. 6d. for the use of the men, their wives, and families; for the custom here is for a subscriber not only to be entitled to a note for himself, as you state, but for his wife or any child living under his roof. They had £63 10s. 1½d. left. Could they, if they thought fit, apply it to purchase tickets to the full value or not? The money was absolutely theirs to divide amongst themselves if by resolution they saw fit. They had entered into contracts by the purchase of tickets with the Dispensary and Hospitals, and their wants had been satisfied. They voted £50 to Hospital Saturday without any conditions attached, and carried forward the balance. Was that or was it not a free gift?

The workshop doctor, as I stated before, is a common institution in Birmingham, and it is generally to supplement his work that dispensary and hospital tickets are purchased. You say:

We are forced to the conclusion, from the figures before us and from those supplied by Mr. Smedley, that as a matter of fact it is probable that the working classes in Birmingham receive more benefits from the hospitals than the sum raised on Hospital Saturday, plus the direct contributions to the hospitals of the working classes, would defray the cost of.

If the working classes provide the entire cost of the hospitals they will cease to be charities in any sense of the word, and will become gigantic provident institutions. The wealthy and middle classes will be relieved from the need of supporting them, and one of the principal channels through which they can perform their obligations to the community will be closed.

If the working classes are to bear the entire cost of the maintenance of the hospitals, the wealthy and middle classes will be receiving benefits of vital importance to them, and allowing the working men to pay the cost for them.

In the footnote to my letter in THE HOSPITAL of May 19th, you say I am

In error, however, in stating that Hospital Saturday in Wolverhampton is managed by the officials, as it is worked by a committee on which there are upwards of fifty working men representatives.

I contend that my statement is absolutely correct. The Wolverhampton and Staffordshire General Hospital has what it terms a Hospital Saturday Committee as a subsidiary committee. It consists of a weekly board of the hospital and 57 working men representatives. This I stated. These are not the officials of the committee. The House Governor and Secretary of the hospital acts as its Secretary. The business is conducted at the hospital by the hospital staff, as a portion of their work. The accounts appear in the annual report of the hospital as a portion of the hospital accounts duly audited by the hospital auditors.

The amounts contributed to the Wolverhampton and Staffordshire Hospital on what is termed Hospital Saturday, in 1892, were—

| | | | Population. |
|---|---|---|---|
| Wolverhampton... | | £1,879 13 6 | 82,620 |
| Bilston, Bradley, and Moxley | | 552 13 6 | 27,580 |
| Cosford ... | | 2 2 0 | — |
| Darlaston | | 229 16 8 | 13,563 |
| Deepfields, Coseley, and Sedgley | | 91 4 1 | 36,860 |
| Dudley ... | | 2 2 0 | 45,740 |
| Ettingshall | | 81 8 4 | 5,763 |
| Heathdown and Wednesfield... | | 63 7 5 | 12,024 |
| Hednesford | | 37 16 0 | 9,020 |
| Swindon ... | | 24 10 7 | 466 |
| Walsall ... | | 23 2 0 | 71,791 |
| Willenhall | | 176 18 11 | 16,852 |
| Wednesbury | | 13 12 7 | 25,342 |
| Sundries | £34 19 11 | | |
| Street ... | 20 14 1 | | |
| | | 55 14 0 | |
| | | £3,234 1 7 | 347,621 |

You have calculated the total of £3,234 on the population of Wolverhampton alone, namely, 82,620, giving £39 per thousand. The actual contribution of Wolverhampton working-men is £1,879 13s. 6d., or £22 12s. per thousand, for which they receive tickets to the same extent as ordinary subscribers. The correct figure for the whole collection is £9 6s. per thousand. There is, in fact, no Wolverhampton Hospital Saturday Fund of the character of the Birmingham Hospital Saturday Fund. WILLIAM T. SMEDLEY.

